# Advances in *Helicobacter pylori* lipopolysaccharide structure and function

**DOI:** 10.1093/femsre/fuaf034

**Published:** 2025-07-26

**Authors:** Xiaoqiong Tang, Alfred Tay, Mohammed Benghezal, Barry J Marshall, Hong Tang, Hong Li

**Affiliations:** Center of Infectious Diseases, West China Hospital, Sichuan University, Chengdu, Sichuan, 610041, China; Laboratory of Infectious and Liver Diseases, Institution of Infectious Diseases, West China Hospital of Sichuan University, Chengdu, Sichuan, 610041, China; Helicobacter pylori Research Laboratory, School of Biomedical Sciences, Marshall Centre for Infectious Disease Research and Training, University of Western Australia, Perth, Nedlands, 6009, Australia; Center of Infectious Diseases, West China Hospital, Sichuan University, Chengdu, Sichuan, 610041, China; Laboratory of Infectious and Liver Diseases, Institution of Infectious Diseases, West China Hospital of Sichuan University, Chengdu, Sichuan, 610041, China; Helicobacter pylori Research Laboratory, School of Biomedical Sciences, Marshall Centre for Infectious Disease Research and Training, University of Western Australia, Perth, Nedlands, 6009, Australia; Center of Infectious Diseases, West China Hospital, Sichuan University, Chengdu, Sichuan, 610041, China; Laboratory of Infectious and Liver Diseases, Institution of Infectious Diseases, West China Hospital of Sichuan University, Chengdu, Sichuan, 610041, China; Center of Infectious Diseases, West China Hospital, Sichuan University, Chengdu, Sichuan, 610041, China; Laboratory of Infectious and Liver Diseases, Institution of Infectious Diseases, West China Hospital of Sichuan University, Chengdu, Sichuan, 610041, China

**Keywords:** *Helicobacter pylori*, lipopolysaccharide, glycosyltransferase, ADP-heptose, colonization, vaccine

## Abstract

*Helicobacter pylori* is a widespread pathogen responsible for chronic gastritis, peptic ulcers, and an elevated risk of gastric cancer. Lipopolysaccharide (LPS), localized exclusively in the outer leaflet of the outer membrane, is essential for maintaining bacterial integrity. Recent advances have deepened our understanding of *H. pylori* LPS structure, particularly lipid A modifications and the redefinition of the core oligosaccharide and O-antigen regions. The complete set of enzymes involved in LPS biosynthesis has been identified in the reference strain G27, and comparative genomics has revealed a notable regional difference (the absence of the heptan domain in East Asian strains). Here, we summarize recent insights into the structure and function of *H. pylori* LPS, emphasizing its role in bacterial persistence and its promise as a target for LPS-based glycoconjugate vaccine development.

## Teaser

Recent insights into *Helicobacter pylori* lipopolysaccharide (LPS) structure and biosynthesis—Including lipid A modifications, redefined core and O-antigen regions, and the absence of heptan in East Asian strains—Underscore its crucial role in bacterial persistence and the implication of regional LPS variations in regional gastric disease risks.

## Introduction

*Helicobacter pylori* is a Gram-negative bacterium that colonizes the gastric mucosal of approximately 40% of the global population (Chen et al. 2024). Upon infection, *H. pylori* adapts to the harsh gastric environment, enabling lifelone colonization unless eradicated by antibiotic treatment (Malfertheiner et al. 2022). Persistent *H. pylori* colonization is strongly associated with chronic gastritis, peptic ulcer disease, and represents the primary etiological factor for gastric cancer (Chey et al. 2024). Nearly 90% of new gastric adenocarcinoma cases are attributable to chronic *H. pylori* infection (Thrift et al. 2023). In recent years, the rising prevalence of antimicrobial resistance in *H. pylori* has posed a significant challenge to effective treatment (Yu et al. 2024). Moreover, the lack of an effective *H. pylori* vaccine, despite decades of research, highlights a critical unmet need in combating this pathogen.

A critical determinant of *H. pylori* survival and pathogenicity within the gastric environment is its lipopolysaccharide (LPS), an essential glycolipid component of the bacterial outer membrane (OM). The OM is an asymmetric structure, with LPS localized in the outer leaflet and phospholipids situated in the inner leaflet. LPS plays a crucial role in maintaining the integrity of the OM and establishes a selective permeability barrier that restricts the entry of antimicrobial agents (Bertani and Ruiz 2018, Sabnis and Edwards 2023, Huang et al. 2024). In addition to its structural role, *H. pylori* LPS modulates host responses by downregulating the pro-regenerative effects of IL-33, which impairs gastric barrier repair (Gonciarz et al. 2021), and by attenuating the antitumor activity of human mononuclear cells (Chochi et al. 2008).

**Figure 1.**
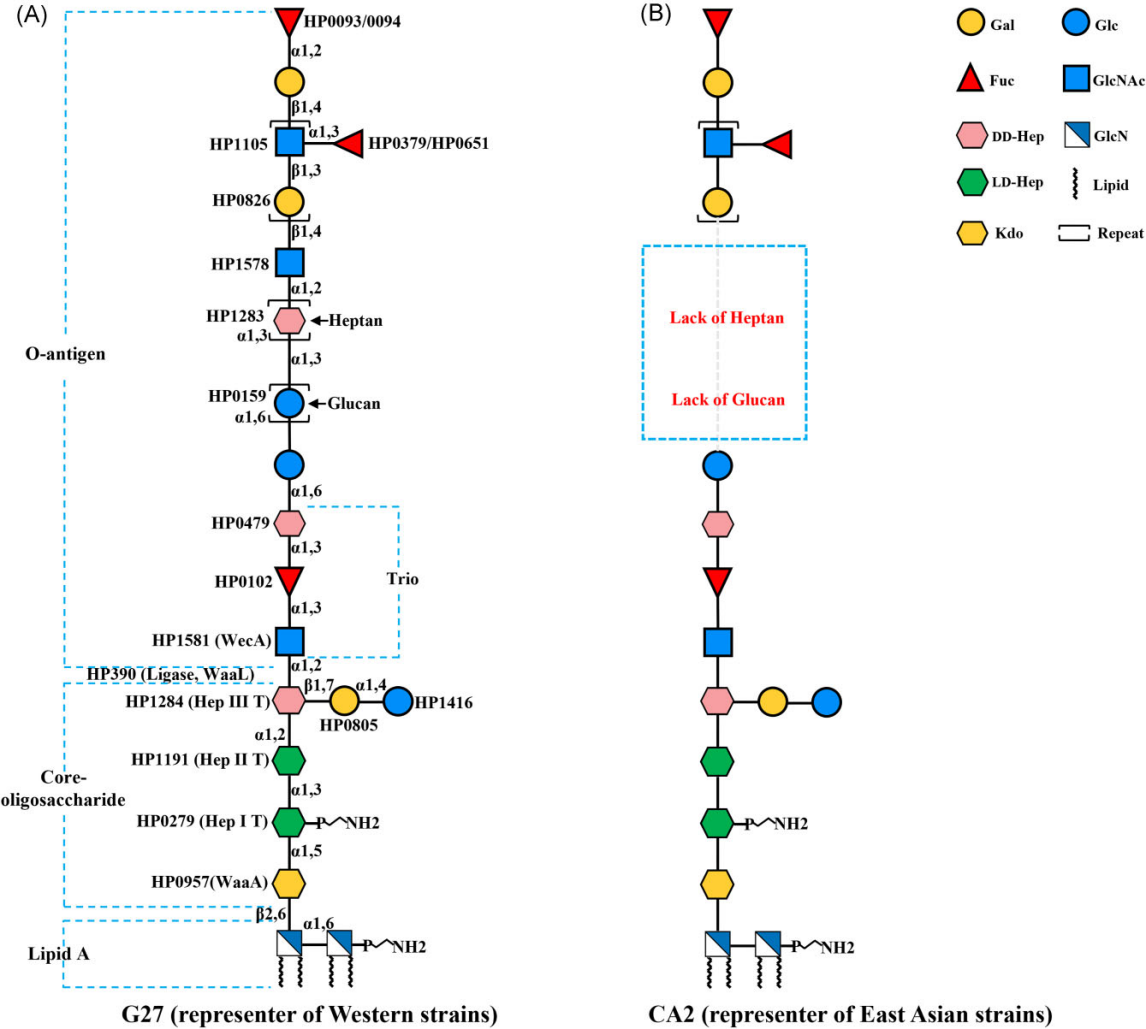
The structural model of *H. pylori* LPS in reference strains G27 and CA2. (A) LPS structure of the reference *H. pylori* strain G27, representing Western *H. pylori* strains. The three regions of LPS are annotated and framed in braces. The glycotransferases involved in LPS synthesis are indicated next to the corresponding glycosyl residues (Li et al. 2017). (B) LPS structure of the reference *H. pylori* strain CA2, representing East Asian *H. pylori* strains (Li et al. 2019). Gal, galactose; Glc, glucose; Fuc, fucose; GlcNAc, *N*-acetyl-glucosamine; DD-Hep, D-*glycero*-D-*mano*-heptose; LD-Hep, L-*glycero*-D-*mano*-heptose; GlcN, glucosamine; Kdo, 3-deoxy-*D*-*manno*-octulosonic acid. Sugar structures are depicted using Symbol Nomenclature for Glycans (SNFG) (Neelamegham et al. 2019, Varki et al. 2015).

Like other Gram-negative bacteria, *H. pylori* LPS is structurally divided into three regions: the hydrophobic lipid A, the intermediate core oligosaccharide, and the terminal O-antigen (Figure 1) (Li et al. 2016). However, *H. pylori* LPS possesses unique characteristics, including low immunogenicity that facilitates immune evasion, and molecular mimicry of host Lewis blood group antigens, which effectively camouflages the bacterium from immune surveillance, thereby promoting persistent colonization (Kadirvelraj et al. 2023). Recent genetic, biochemical, and structural studies have refined our understanding of *H. pylori* LPS structure, biosynthesis, and modification, highlighting its important roles in bacterial physiology and pathogenesis. This review updates current knowledge regarding *H. pylori* LPS, focusing on lipid A modification for immune evasion; the structural redefinition of the core oligosaccharide and O-antigen regions and their roles in host colonization; LPS structural differences between Western and East Asian *H. pylori* strains; and the role of ADP-heptose (ADP-Hep, the precursor of Hep units present in the core oligosaccharide and O-antigen regions) as a novel pathogen-associated molecular pattern (PAMP). Finally, we discuss the potential for developing LPS-based conjugate vaccines.

## The unique structure of *H. pylori* lipid A is crucial for the bacterium's immune evasion

The typical lipid A structure of most Gram-negative bacteria comprises of a β-(1→6)-linked glucosamine (GlcN) disaccharide backbone, with two negatively-charged phosphate groups attached at the 1 and 4' positions (referred as double phosphorylation), two negatively-charged Kdo (3-deoxy-D-*manno*-oct-2-ulosonic acid) residues, and six acyl chains (typically 12–14 carbons in length) esterified or amide-linked to the backbone (Ma et al. 2017, Hofstaedter et al. 2024). This canonical lipid A structure acts as a potent activator of Toll-like receptor 4 (TLR4), triggering robust innate immune responses, including the production of inflammatory cytokines and cationic antimicrobial peptides (CAMPs) (Aderem and Ulevitch 2000). CAMPs bind to the negatively charged lipid A, disrupting membrane integrity and inducing bacterial lysis (Diamond et al. 2009). While this innate immune response can aid the host in clearing invading patogens, excessive lipid A-induced inflammatory response can lead to severe pathological conditions, such as septic shock, multi-organ failure, and even death (Arbour et al. 2000, Kuzmich et al. 2017, Slomiany and Slomiany 2017). Due to its potent biological activity and toxicity, lipid A is recognized as endotoxin in most Gram-negative bacteria.

**Figure 2.**
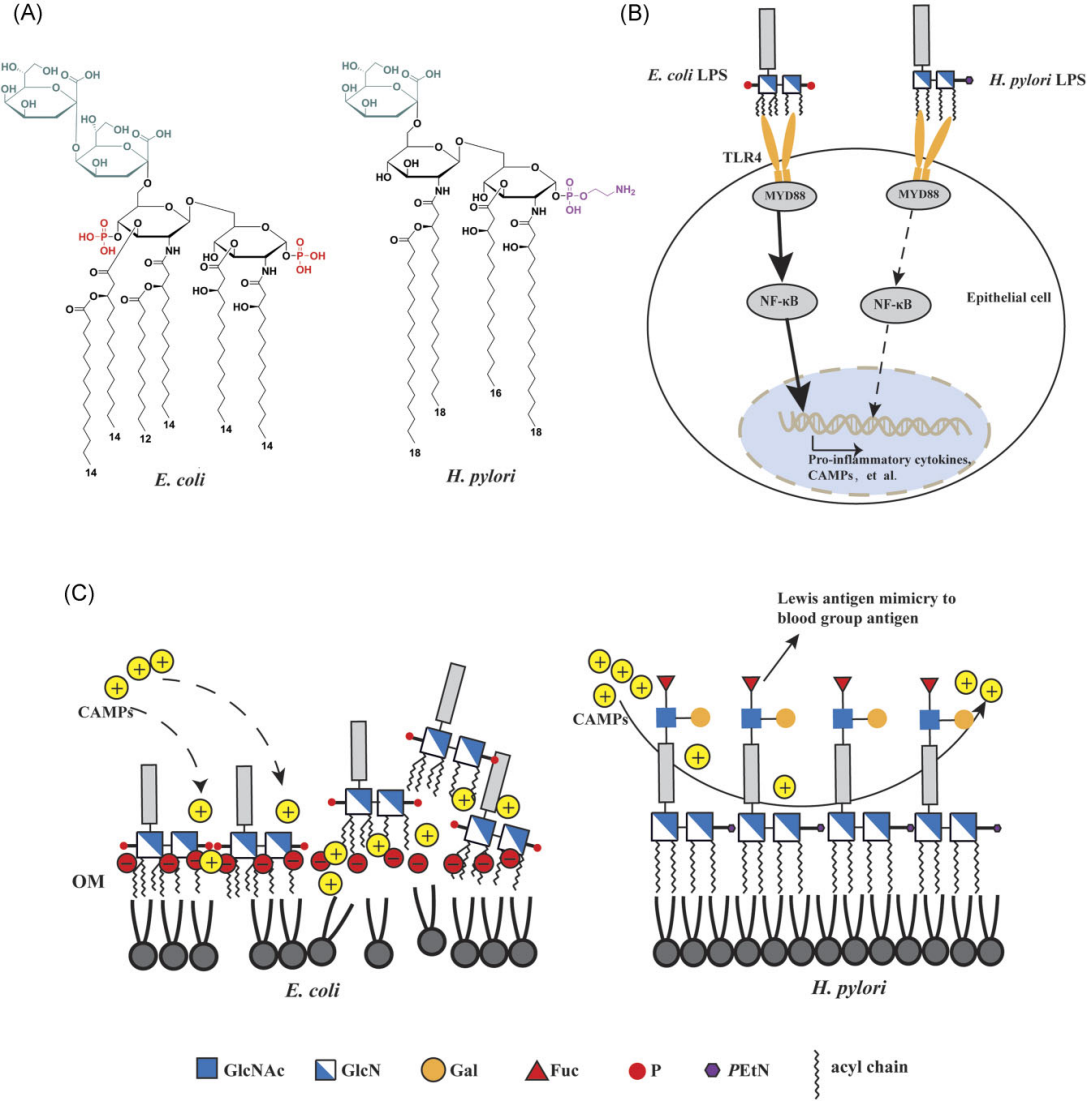
Molecular mechanisms of *H. pylori* LPS-mediated immune evasion and colonization. (A) Structural comparison of the lipid A-Kdo between *E. coli* and *H. pylori* (Cullen et al. 2011, Ma et al. 2017). (B) Differential TLR4 activation: *E. coli* lipid A (bis-phosphorylated hexa-acylated with C12-C14 chains) strongly activates TLR4, inducing robust inflammatory cytokine and CAMP production that mediates bacterial clearance. In contrast, *H. pylori* lipid A (mono-PEtN modified tetra-acylated with C18 chains) exhibits weak TLR4 agonism, resulting in attenuated immune responses that permit persistent colonization (Cullen et al. 2011). (C) Evasion strategies: (1) lipid A modifications (reduced phosphate groups and elongated acyl chains) decrease lipid A electronegativity, preventing CAMP binding and membrane disruption; (2) terminal Lewis antigen mimicry of host blood group antigens, hindering host immune recognition. Kdo, 3-deoxy-D-*manno*-octulosonic acid; TLR4, toll-like receptor 4; GlcNAc, N-acetylglucosamine; GlcN, glucosamine; Gal, galactose; Fuc, fucose; P, phosphate group; *P*EtN, phosphoethanolamine; CAMP, cationic antimicrobial peptide.

Unlike the canonical lipid A structure in most Gram-negative bacteria, *H. pylori* lipid A undergones constitutive modifications that result in: 1) removal of the negatively charged phosphate groups at the 1 and 4' positions of the GlcN disaccharide backbone, with the 1-position phosphate being replaced by a phosphoethanolamine (PEtN) moiety; 2) removal of one Kdo residue, leaving only a single Kdo; and 3) reduction from six to four acyl chains through deacylation (Mattsby-Baltzer et al. 1992, Moran et al. 1997, Tran et al. 2004, Tran et al. 2006, Cullen et al. 2011). Notably, the acyl chains in *H. pylori* lipid A are 16–18 carbon atoms in length, which is distinctly different from the 12–14 carbon acyl chains predominant in most Gram-negative bacteria (Cullen et al. 2011) (Figure 2A).

The distinctive dephosphorylated, tetra-acylated, mono-Kdo configuration of *H. pylori* lipid A significantly reduces its capacity as a TLR4 agonist, resulting in immunostimulatory activity that is over 1000-fold weaker than that of canonical enterobacterial lipid A (Maldonado et al. 2016) (Figure 2B). This unique lipid A structure also confers *H. pylori* natural resistance to CAMPs, due to the loss of negatively charged phosphate groups and a Kdo residue (Figure 2C). Notably, *H. pylori* resists calprotectin, an antimicrobial protein released by activated neutrophils during inflammation (Gaddy et al. 2015). This resistance is achieved by enhancing bacterial fitness and biofilm formation through the inhibition of constitutive lipid A modification enzymes (LpxF, LpxL, and LpxR) (Gaddy et al. 201). The inhibition of these enzymes leads to insufficient lipid A dephosphorylation and deacylation, which could potentially increase *H. pylori*’s susceptibility to TLR4 recognition and subsequent elimination. However, the insufficiently modified lipid A binds more efficiently to host annexins, effectively shielding the phosphorylated lipid A molecules from TLR4 recognition (Schmidinger et al. 2022). Consequently, constitutive modifications of *H. pylori* lipid A, and the modulation of these modifications under stress, are crucial for the bacterium to establish persistent colonization within the gastric environment (Cullen et al. 2011). This is likely a result of adaptation to the human stomach over 100 000 years of co-evolution (Atherton and Blaser 2009, Montano et al. 2015).

**Figure 3.**
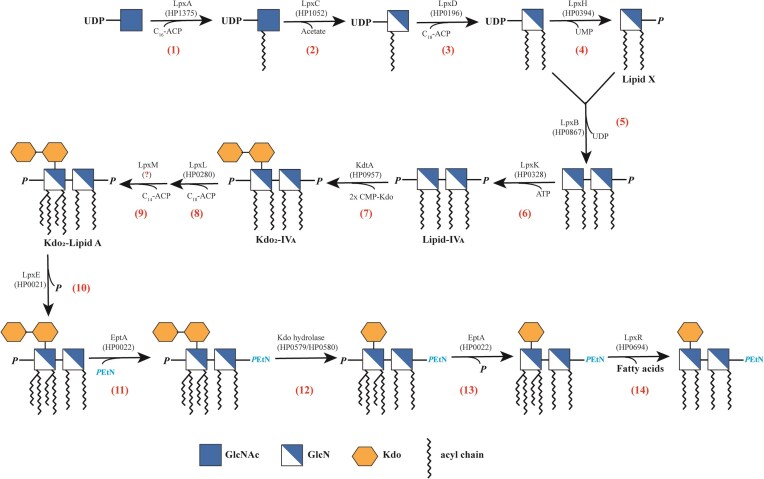
The conserved lipid A biosynthesis and constitutive modification pathway in *H. pylori*. The whole pathway comprises fourteen enzymatic steps: nine for biosynthesis (eight Lpx enzymes and one KdtA glycosyltransferase) and five for modification (three Lpx enzymes, one PEtN transferase, and one Kdo hydrolase) (Raetz and Whitfield 2002, Stead et al. 2005, Stead et al. 2010). Step 1: LpxA (HP1375) adds an acyl chain to UDP-GlcNAc. Step 2: LpxC (HP1052) removes the acetyl group from UDP-3-O-acyl-GlcNAc. Step 3: LpxD (HP0196) adds a second acyl chain to form UDP-2,3-diacyl-GlcN. Step 4: LpxH (HP0394) cleaves the pyrophosphate bond, producing lipid X. Step 5: LpxB (HP0867) condenses lipid X with UDP-2,3-diacyl-GlcN, forming the tetra-acyl-disaccharide-1-phosphate. Step 6: LpxK (HP0328) phosphorylates the molecule at the 4'-position, producing lipid-IV_A_. Step 7: KdtA (HP0957) adds two Kdo sugars from CMP-Kdo to lipid-IVA, generating Kdo2-IV_A_. Step 8: LpxL (HP0280) adds a secondary acyl chain to the 2'-position of Kdo2-IV_A_. Step 9: LpxM (unidentified homologue in *H. pylori*) adds a secondary acyl chain to the 3'-position, completing the biosynthesis. Step 10: LpxE (HP0021) removes the 1'-phosphate group from Kdo2-lipid A. Step 11: EptA (HP0022) adds a *P*EtN group to the 1-position of Kdo2-lipid A. Step 12: KdoH1 (HP0579) and KdoH2 (HP0580) work together to cleave off the terminal Kdo sugar. Step 13: LpxF (HP1580) removes the 4'-phosphate group, leaving a hydroxyl group. Step 14: LpxR (HP0694) removes the 3-O-linked acyl chains, resulting in a tetra-acylated lipid A.

The *de novo* biosynthesis of *H. pylori* lipid A follows the canonical Raetz pathway, which comprises nine steps that involve eight Lpx enzymes (LpxA, LpxB, LpxC, LpxD, LpxH, LpxK, LpxL, LpxM) and one Kdo glycosyltransferase (KdtA) (Raetz and Whitfield 2002, Stead et al. 2005, Stead et al. 2010, Li et al. 2016). The constitutive lipid A modification pathway has been elucidated as a strictly ordered five-step process involving five enzymes (the lipid A 1-phosphatase LpxE, the lipid A 4-phosphatase LpxF, the *P*EtN transferase EptA, the Kdo hydrolase KdoH, and the deacylase LpxR) (Cullen et al. 2011, Stead et al. 2010, Tran et al. 2004, Tran et al. 2006) (Figure 3).

## The redefinition of *H. pylori* LPS core oligosaccharide and O-antigen regions

The polysaccharide structure of *H. pylori* LPS can be devided into the core oligosaccharide and O-antigen regions. The precise distinction between these two domains relies on their structural characterization and the understanding of the corresponding biosynthetic pathways.

Early structural investigations indicated that, similar to other Gram-negative bacteria, the core oligosaccharide structure of *H. pylori* LPS is organized into a classical inner core and outer core, while its O-antigen is comprised solely of Lewis antigens (Altman et al. 2011, Li et al. 2016). Specifically, in *H. pylori* strain 26 695, the initially assigned inner core was postulated to consist of a conserved hexasaccharide (Glc-Gal-DD-Hep-LD-Hep-LD-Hep-KDO), and the outer core was postulated to include a DD-heptan with the first DD-Hep residue linked to a side-branched α-1,6-glucan(Altman et al. 2008, Hiratsuka et al. 2005, Logan et al. 2005, Logan et al. 2000, Monteiro et al. 2000). Subsequent reinvestigation, however, revealed the outer core to be a linear structure composed of a trisaccharide (GlcNAc-Fuc-DD-Hep), DD-heptan, and α-1,6-glucan (Altman et al. 2011). Notably, because the O-antigen ligase and many glycosyltransferases involved in the biosynthesis of these polysaccharide structures remained unidentified at the time, the distinction between the core oligosaccharide and O-antigen regions was largely conceptual.

To precisely define the the core oligosaccharide and O-antigen regions of *H. pylori* LPS, our group constructed the O-antigen ligase mutant Δ*waaL* in the reference strain G27 (Li et al. 2017). This strain is fully sequenced, extensively used in *H. pylori* research, and genetically easy to manipulate in the laboratory (Baltrus et al. 2009). Highly pure LPS samples isolated from G27 wild-type and G27Δ*waaL* were structurally analyzed by mass spectrometry (MS) and nuclear magnetic resonance (NMR) spectroscopy (Li et al. 2017). The analysis revealed that the LPS structures of G27 and strain 26 695 are nearly identical. Notably, the core oligosaccharide structure in G27Δ*waaL*, lacking the whole O-antigen, was revealed to be a short, conserved hexasaccharide, lacking the canonical inner and outer core organization. Consequently, the O-antigen of *H. pylori* LPS was redefined as a very long structure, encompassing not only Lewis antigens, but also the Trio, DD-heptan, and α-1,6-glucan (previously defined as the outer core) (Li et al. 2017) (Figure 1A).

Following the redefinition of the LPS core oligosaccharide and O-antigen regions, our group conducted a genome-wide search for glycosyltransferase genes in *H. pylori* strain G27 using the Carbohydrate-Active Enzymes (CAZy) database (Li et al. 2019). This was followed by a systematic mutational analysis of LPS genes in G27 using the Xer-cise gene deletion technique developed by our group. Subsequently, highly pure LPS samples were purified from the constructed LPS mutants for structural characterization and comparison with the wild-type LPS structure. This approach enabled our group to successfully identify the Hep-III transferase HP1284, the Trio Fuc transferase HP0102, the heptan transferase HP1283, and the GlcNAc transferase HP1578, which initiates the synthesis of Lewis antigens onto the DD-heptan motif (Li et al. 2019). Furthermore, another glycosyltransferase, HP0805, is deduced to transfer the Gal residue to Hep-III in the core oligosaccharide (Li et al. 2019). The identification of these previously unidentified glycosyltransferase genes facilitated the establishment of the first complete LPS biosynthetic pathway in G27 (Figure 1A, Table 1).

**Table 1.** The complete set of glycosyltransferases involved in LPS core-oligosaccharide and O-antigen biosynthesis of *H. pylori* reference strain G27.

Domains		GTs	Demonstrated functions	References
Core-oligosaccharide		HP0279	HepI transferase for assembling core-oligosaccharide	(Mobley et al. 2001)
		HP1191	HepII transferase for assembling core-oligosaccharide	(Chandan et al. 2007, Li et al. 2019)
		HP1284	HepIII transferase for assembling core-oligosaccharide	(Altman et al. 2018, Li et al. 2019, Li et al. 2017)
		HP0805	Gal transferase for assembling the core-oligosaccharide	(Li et al. 2019)
		HP1416	Glc transferase for assembling the core-oligosaccharide	(Li et al. 2019, Moran et al. 2004)
O-antigen	Trio	HP1581	GlcNAc transferase for the initiation of O-antigen	(Hug et al. 2010, Li et al. 2019)
		HP0102	Fuc transferase for assembling the Trio motif	(Li et al. 2019, Pernitzsch et al. 2021)
		HP0479	Hep transferase for assembling the Trio motif	(Hiratsuka et al. 2005, Li et al. 2019)
	Glucan	HP0159	Glc transferase for assembling the glucan structure	(Altman et al. 2008, Moran et al. 2004)
	Heptan	HP1283	Hep transferase for assembling the DD-heptan structure	(Altman et al. 2018, Li et al. 2019)
	Lewis antigen	HP1578	Adding the first GlcNAc to the heptan for the LacNAc initiation	(Li et al. 2019)
		HP0826	β-(1,4)-Gal transferase for assembling the Lewis antigen	(Altman et al. 2011, Li et al. 2019)
		HP1105	β-(1,3)-GlcNAc transferase for assembling the Lewis antigen	(Li et al. 2019, Logan et al. 2005)
		HP0379	FutA, α-(1,3/4)-Fuc transferase for assembling the Lewis antigen	(Li et al. 2019, Ma et al. 2006, Nilsson et al. 2006)
		HP0651	FutB, α-(1,3/4)-Fuc transferase for assembling the Lewis antigen	(Li et al. 2019, Ma et al. 2006, Nilsson et al. 2006)
		HP0093/0094	FutC, α-(1,2)-Fuc transferase for assembling the Lewis antigen	(Li et al. 2019, Wang et al. 1999)

GTs, glycosyltransferases; Hep, heptose; Gal, galactose; Glc, glucose; GlcNAc, *N*-acetyl-glucosamine; Fuc, fucose; LacNAc, N-acetyllactosamine.

**Figure 4.**
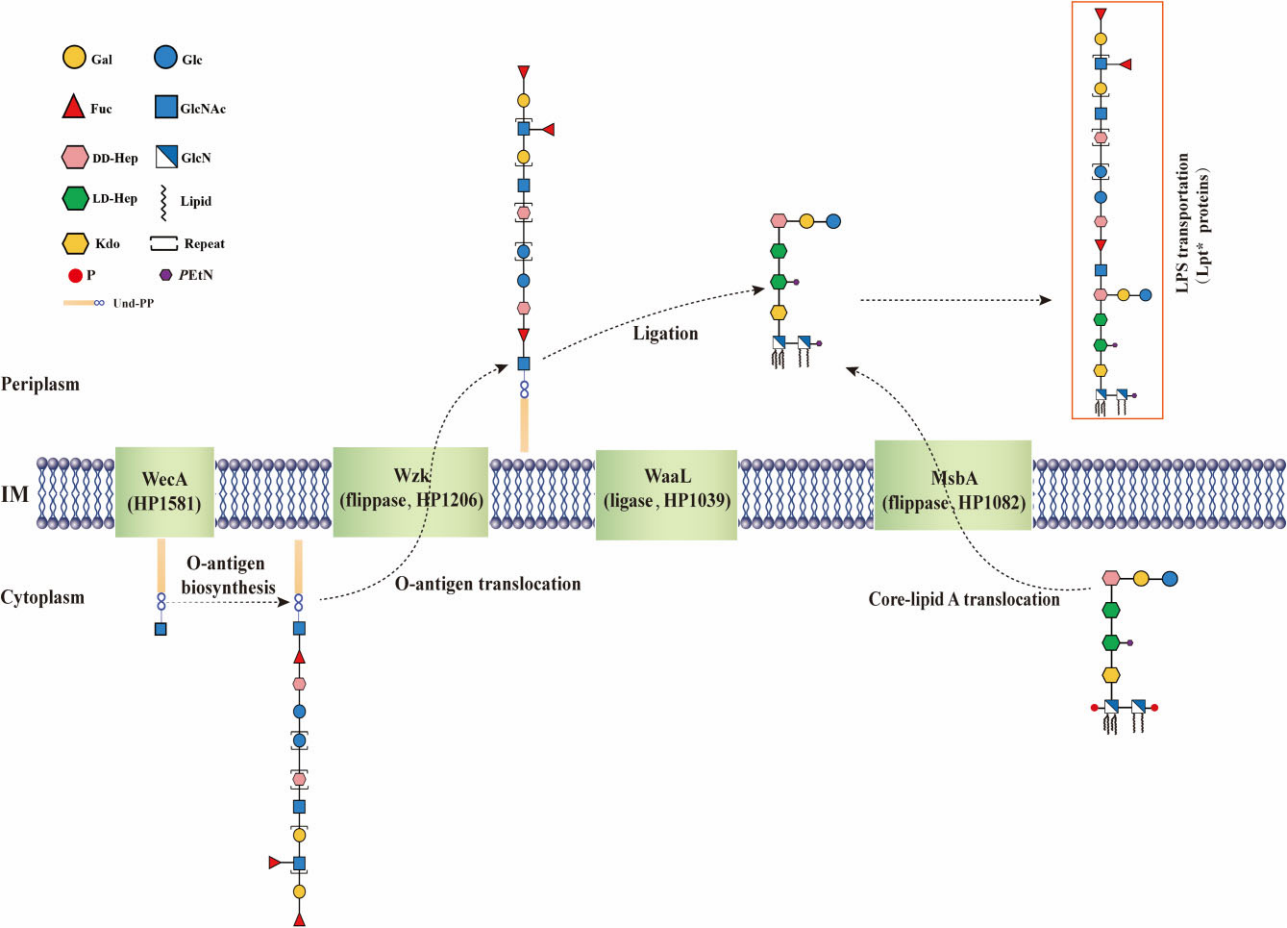
*H. pylori* LPS biosynthesis and transport. WecA (HP1581) initiates O-antigen biosynthesis by transferring GlcNAc to Und-PP carrier. Sequential glycosyltransferases then elongate the O-antigen with specific monosaccharides. The completed O-antigen is flipped across the inner membrane by Wzk (HP1206). Core-lipid A, synthesized in the cytoplasm and flipped by MsbA (HP1082), is ligated to the O-antigen by WaaL (HP1039) in the periplasm to form mature LPS (Chiu et al. 2009, Li et al. 2017). The Lpt transport system mediates LPS transit across the periplasm for final insertion into the outer membrane. Gal, galactose; Glc, glucose; Fuc, fucose; GlcNAc, *N*-acetyl-glucosamine; DD-Hep, D-*glycero*-D-*mano*-heptose; LD-Hep, L-*glycero*-D-*mano*-heptose; GlcN, glucosamine; Kdo, 3-deoxy-*D*-*manno*-octulosonic acid; Und-PP, undecaprenyl phospholipid; IM, inner membrane; OM, outer membrane.

The redefinition of the LPS core oligosaccharide and O-antigen regions, combined with the identification of previously unkown glycosyltransferase genes involved in *H. pylori* polysaccharide biosynthesis, provides new insights into both the structural conservation and variability of *H. pylori* LPS regions and their associated biosynthetic and transport pathways (Figure 4, Table 1). The biosynthesis of the long O-antigen and lipid A-core occurs separately in the cytoplasm. Notably, WecA initiates O-antigen biosynthesis by transferring a GlcNAc moiety onto a undecaprenyl phospholipid (UndPP) carrier in the cytoplasm, thereby catalyzing the assembly of the GlcNAc residue in the Trio structure. Upon completion of their synthesis, they are translocated across the inner membrane to the periplasm by the flippases Wzk and MsbA, respectively (Chiu et al. 2009, Hug et al. 2010). In the periplasm, the O-antigen is ligated to the Lipid A-core by the ligase WaaL to form the full-length LPS structure, which is subsequently transported to the cell surface by the LPS transport (Lpt) system (Li et al. 2017). While the Lpt system has been extensively characterized in *E. coli*, it is generally conserved in Gram-negative bacteria (Bos and Tommassen 2011, Martorana et al. 2016). The *E. coli* Lpt system spans the inner and OMs, involving several key proteins: LptB (cytoplasm), LptF, LptG, and LptC (inner membrane), LptA (periplasm), and LptD and LptE (OM) (Villa et al. 2013, Putker et al. 2015). *H. pylori* possesses homologs of most *E. coli* Lpt proteins, suggesting a similar LPS transport mechanism (Liechti and Goldberg 2012). However, the precise mechanisms of LPS transport in *H. pylori*, including the specific roles of each Lpt protein and potential unique adaptations, remain to be elucidated.

## Core oligosaccharide and Trio structures are highly conserved and essential for *H. pylori* colonization

Using the complete LPS glycosyltransferase gene set of *H. pylori* strain G27 as a reference, comparative genomic analysis of 177 diverse *H. pylori* strains revealed that the all glycosyltransferase genes involved in the synthesis of core oligosaccharide and Trio are conserved (Li et al. 2019). This conservation provides genetic evidence for the structural preservation of these moieties across strains. In contrast, the heptan transferase gene *HP1283* exhibits high variability among strains of different phylogeographic origin (Li et al. 2019). Notably, clinical isolates lacking both heptan and Lewis antigens, as well as the Δ*HP0159* mutant (truncated from glucan) and Δ*HP0826* mutant (Lewis antigen-truncated), retained the ability to colonize the host (Altman et al. 2003, Altman et al. 2008, Chandan et al. 2013), suggesting that these structures are dispensable for colonization. However, the Δ*waaL* mutant (lacking the entire O-antigen), Δ*HP0102* mutant (truncation from the Trio Fuc residue), Δ*HP0479* mutant (truncation from the Trio Hep residue), and Δ*HP1284* mutant (loss of Hep-III and the attached disaccharide) all failed to colonize in a mouse stomach model (Hiratsuka et al. 2005, Li et al. 2017, Pernitzsch et al. 2021). This suggests that the core oligosaccharide and Trio structures are essential for colonization.

The critical role of the LPS core oligosaccharide and Trio structures in *H. pylori* colonization may be partly ascribed to their functions in maintaining *H. pylori* spiral morphology, conferring resistance to CAMPs (Tang et al. 2023), and binding with host annexins (Schmidinger et al. 2022). Deletion of key glycosyltransferase genes resulted in altered bacterial morphology, particularly evident after 48 hours of culture: while wild-type G27 retained its spiral shape, the Δ*wecA* mutant transitioned almost entirely to a coccoid form. Polymyxin B susceptibility assays showed that Δ*HP1578* (Lewis antigen-deficient) and Δ*HP1283* (heptan- and Lewis antigen-deficient) mutants had MIC values comparable to wild-type strains. In contrast, Trio mutants (Δ*wecA*, Δ*HP0479*, Δ*HP0102*) and core oligosaccharide mutants (Δ*HP1191*, Δ*HP1284*) exhibited significantly increased polymyxin B sensitivity, with up to a 60-fold reduction in MIC (Pernitzsch et al. 2021, Tang et al. 2023). Moreover, the core oligosaccharide, specifically the Hep III and the attached disaccharide, along with lipid A serves as a ligand for host annexin binding. This interaction plays an important role in modulating TLR4 recognition and host colonization (Schmidinger et al. 2022).

## Lewis antigen mimicry: a double-edged sword of immune evasion and autoimmunity

Lewis antigens, built upon a Gal-GlcNAc backbone, are classified into two types based on glycosidic linkage (Logan et al. 2005, tman et al. 2011, Li et al. 2019). Type 1 Lewis antigens are characterized by a Gal-(β-1,3)-GlcNAc linkage, formed by the β-1,3-Gal transferase JHP0563 (Pohl et al. 2009, hl et al. 2012), while Type 2 Lewis antigens possess a Gal-(β-1,4)-GlcNAc linkage, synthesized by the β-1,4-Gal transferase HP0826 (Altman et al. 2011). Fucosylation of the GlcNAc residue within Type I and Type II chains by the α-1,3-Fuc transferases HP0379/HP0651 (FutA/FutB) yields Lewis a (Le^a^) and Lewis x (Le^x^), respectively (Ma et al. 2006, Nilsson et al. 2006, Li et al. 2019). Subsequent terminal fucosylation of Le^a^ or Le^x^ by the α-1,2-Fuc transferase HP0093/0094 (FutC) generates difucosylated Lewis b (Le^b^) or Lewis y (Le^y^), respectively (Wang et al. 1999, i et al. 2019 (Table 1, Figure 1A). Type 2 Le^x/y^ antigens are prevalent in 80–90% of *H. pylori* strains globally, while Type 1 Le^a/b^ antigens are more frequently expressed in Asian strains (Pohl et al. 2011, Tang et al. 2023). Structural analyses have revealed the simultaneous expression of both type 1 and type 2 regions on the same O-chain of the same strain, with Type 1 antigens frequently capping Type 2 chains (Monteiro et al. 1998).

Comparative genomic analysis of 177 diverse *H. pylori* strains has revealed a high degree of conservation in the glycosyltransferase genes involved in Lewis antigen biosynthesis (Li et al. 2019). This highlights the importance of Lewis antigen expression for *H. pylori*’s adaptation and persistence within the host gastric mucosa. While the genes themselves are conserved, frameshift mutations within homopolymeric tracts are commonly observed in the three fucosyltransferase genes (HP0379/HP0651/HP0093/0094), leading to the on/off switching of these enzymes and, consequently, phase variation of Lewis antigen expression (Li et al. 2019). This allows *H. pylori* to tailor its Lewis antigen phenotype to match that of its host, enabling immune evasion (Figure 2B). This is exemplified by the observation that when Type I Le^b^-expressing transgenic mice were challenged with an *H. pylori* strain expressing Type II Le^x/y^, bacterial populations recovered after 8 months of infection primarily expressed Leb, with a concomitant loss of Type II Le^x/y^ expression (Pohl et al. 2009).

Beyond immune evasion, *H. pylori*’s Lewis antigen mimicry has implications for autoimmune diseases, particularly atrophic gastritis (Appelmelk et al. 1996, Negrini et al. 1996, Negrini et al. 1997, Annibale et al. 2001). By expressing Lewis antigens similar to those found on host cells (e.g. gastric epithelial cells, blood cells, and secretory cells), *H. pylori* can trigger the production of autoantibodies (Appelmelk et al. 1996, Negrini et al. 1997). These autoantibodies, initially targeting the bacteria, may subsequently cross-react with host cells expressing similar Lewis antigens. For instance, anti-Lewis antibodies can react with gastric mucin, contributing to inflammation and tissue damage. Moreover, anti-Lex antibodies can target polymorphonuclear leukocytes (PMNs), and anti-Ley antibodies can target the H+, K+-ATPase on gastric parietal cells, leading to autoimmune destruction and contributing to the development of atrophic gastritis (Appelmelk et al. 1996). Therefore, Lewis antigen mimicry, while facilitating immune evasion, can paradoxically also induce autoimmune gastric diseases.

## The presence or absence of DD-heptan represents a key difference in LPS structure between Western and East Asian *H. pylori* strains

Heptose, a 7-carbon carbohydrate not produced by mammals, is a conserved component of the LPS core oligosaccharide in Gram-negative bacteria (García-Weber and Arrieumerlou 2020). While most Gram-negative bacteria, including *E. coli* and *Salmonella*, predominantly incorporate L-*glycero*-D-*manno*-heptose (LD-Hep) into their LPS core structures, D-*glycero*-D-*manno*-heptose (DD-Hep) is rarely utilized (Valvano et al. 2000, Kneidinger et al. 2002, Kim 2003). Interestingly, *H. pylori* displays a unique LPS architecture that incorporates both LD- and DD-heptose residues within its core oligosaccharide and O-antigen regions (Li et al. 2017). Specifically, the Hep-I and Hep-II residues are LD-Hep moieties transferred by heptosyltransferases HP0279 and HP1191, respectively, while the Hep-III and Trio Hep residues consist of DD-Hep transferred by HP1284 and HP0479 (Table 1). Notably, Western *H. pylori* strains such as 26 695, G27, O:3, O:6 and MO19 commonly contain an extended DD-heptan structure composed of more than 20 DD-heptose residues (Aspinall and Monteiro 1996, Aspinall et al. 1997, Altman et al. 2011, Li et al. 2017). In contrast, LPS structural analysis of 12 East Asian strains (4 Chinese, 5 Japanese and 3 Singaporean isolates) revealed a complete absence of this DD-heptan moiety (Monteiro et al. 2000) (Figure 1B). Consistent with this finding, our group identified that HP1283 (encoding a heptan transferase) (Shih et al. 2001) and HP1578 (encoding a GlcNAc transferase responsible for initiating Lewis antigen synthesis onto the heptan) were entirely absent in all 74 East Asian strains examined (Li et al. 2019). In contrast, these genes were present in ∼80% of European strains (47/59). This genomic divergence aligns with the observed LPS structural differences between Western and East Asian *H. pylori* strains.

Considering that East Asian strains lack the heptan structure but still express terminal Lewis antigens, we investigated how the Lewis antigens are linked to the conserved Glc-Trio-Core. Notably, East Asian strains lacking HP1283/HP1578 commonly feature two copies of HP1105 (encoding a GlcNAc transferase involved in Lewis antigen synthesis) and JHP0562 (encoding a Gal transferase also involved in Lewis antigen synthesis). In contrast, Western strains containing HP1283/HP1578 generally have only one HP1105 copy and lack JHP0562 (Li et al. 2019). This genetic variation implies that the additional HP1105 and the presence of JHP0562 in East Asian strains might substitute for the absence of HP1283/HP1578, allowing Lewis antigens to attach directly to the Glc-Trio-Core. We hypothesize that Lewis antigen construction in East Asian strains begins with either the supplementary HP1105 or JHP0562, transferring GlcNAc or Gal, respectively, to the Glc-Trio-Core. Our lab has experimentally confirmed this model using a G27 strain background: knock-out of *HP1283/HP1578* abolished heptan-dependent Lewis antigen synthesis, but subsequent knock-in the additional HP1105 or JHP0562 restored Lewis antigen expression (unpublished data).

Since DD-Hep is rarely utilized by most other Gram-negative bacteria, its common presence in the LPS of Western *H. pylori* strains is intriguing. This raises an important question: does the presence or absence of DD-heptan in Western versus East Asian *H. pylori* strains contribute to differences in virulence? Previous studies have suggested that the heptan moiety might serve as a structural extension, increasing LPS length and flexibility, thereby enhancing the exposure of Lewis antigens (Altman et al. 2003). This could facilitate molecular mimicry, allowing for immune evasion.

Considering that *H. pylori* infection is a primary risk factor for gastric cancer—a disease disproportionately prevalent in East Asia (Bray et al. 2024)– the absence of DD-heptan in the LPS of East Asian strains represents a significant structural distinction that may play a role in strain-specific virulence and host-pathogen interactions.

## ADP-Hep, the precursor for LPS heptose residues, is a novel PAMP in *H. pylori*

**Figure 5.**
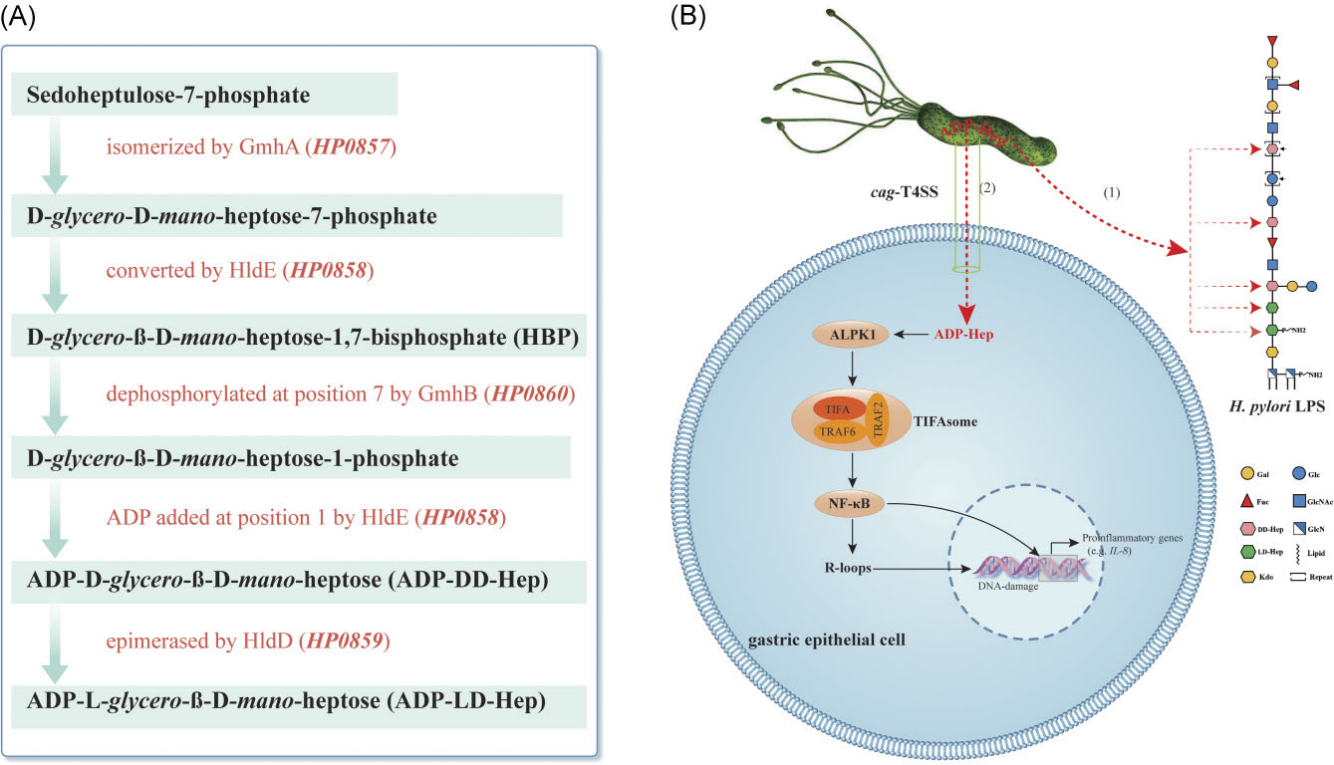
Biosynthetic pathway and biological roles of ADP-Hep. (A) The five-step biosynthetic pathway of ADP-Hep (including ADP-DD-Hep and ADP-LD-Hep). Metabolites are shown in black. Enzymes and their corresponding genes (from *H. pylori* reference strain 26 695) are indicated in red (Chiu et al. 2021, Shaik et al. 2011, Yu et al. 2016). (B) Cellular fates of ADP-Hep: (1) serves as the donor of Hep residues for LPS synthesis; (2) functions as pathogen-associated molecular pattern (PAMP) that is translocated via the *cag* type 4 secretion system (*cag*-T4SS) into host cells, inducing inflammatory responses and DNA damage (García-Weber and Arrieumerlou 2020). ADP-Hep, adenosine diphosphate heptose; ALPK1, alpha kinase 1; TRAF, tumor necrosis factor receptor-associated factor; TIFA, TRAF-interacting protein with a forkhead-associated domain; TIFAsome, TIFA complexes with TRAFs; NF-κB, nuclear factor kappa-B; R-loops, co-transcriptional RNA-DNA hybrids.

The nuceotide activated sugars ADP-LD-Hep and ADP-DD-Hep are precursors for the incorporation of LD-Hep and DD-Hep residues, respectively, into the *H. pylori* LPS structure (Li et al. 2016). The biosynthesis of ADP-LD-Hep proceeds through a five-step enzymatic pathway involving isomerase, kinase, phosphatase, nucleotidyltransferase, and epimerase activities (Kneidinger et al. 2002, Valvano et al. 2002, García-Weber and Arrieumerlou 2020). In *H. pylori*, the genes encoding these enzymes are organized in a genomic cluster (*HP0857* to *HP0860*) (Tomb et al. 1997, Shaik et al. 2011, Yu et al. 2016, Chiu et al. 2021) (Figure 5A). The biosynthetic pathway initiates with sedoheptulose-7-phosphate (S7P), which is converted to D-*glycero*-D-*manno*-heptose-7-phosphate (H7P) by the isomerase GmhA (HP0857) (Yu et al. 2016). The kinase domain of the bifunctional enzyme HldE (HP0858) then phosphorylates H7P to produce D-*glycero*-β-D-*manno*-heptose-1,7-bisphosphate (HBP) (Zimmermann et al. 2017). Subsequent dephosphorylation by the phosphatase GmhB (HP0860) yields D-*glycero*-β-D-*manno*-heptose-1-phosphate (H1P) (Chiu et al. 2021). The nucleotidyltransferase domain of HldE (HP0858) subsequently catalyzes the transfer of an adenylyl group to H1P, generating ADP-DD-Hep. Finally, the epimerase HldD (HP0859) converts ADP-DD-Hep into ADP-LD-Hep (Shaik et al. 2011).

Both H1P and HBP were initially identified as activators of NF-kB-mediated immune responses, with HBP proposed as a novel pathogen-associated molecular pattern (PAMP) (Malott et al. 2013, Gaudet et al. 2015, Gall et al. 2017, Stein et al. 2017, Zimmermann et al. 2017). However, Zhou *et al*. demonstrated that ADP-Hep (including both ADP-DD-Hep and ADP-LD-Hep), rather than HBP, enters the host cytosol to activate NF-κB (Zhou et al. 2018). In *Yersinia pseudotuberculosis*, ADP-Hep relies on the type III secretion system (T3SS) for cytosolic delivery, where it directly binds the N-terminal domain of alpha kinase 1 (ALPK1) (Zhou et al. 2018). This interaction stimulates the kinase domain of ALPK1 to phosphorylate and activate TRAF-interacting protein with a forkhead-associated domain (TIFA), triggering downstream immune signaling. Notably, a very recent study revealed that certain heptose nucleotidyltransferases—such as those from *Burkholderia multivorans* or *B. cepacia*—possess a signature STT_R5_ motif characterized by an arginine residue at the fifth position of the consensus motif (F/L)XXGXSTT (Tang et al. 2024). This structural feature confers NTP substrate promiscuity, enabling the synthesis of CDP-Hep or UDP-Hep in addition to ADP-Hep. Intriguingly, both CDP-Hep and UDP-Hep induce stronger ALPK1-TIFA-mediated immune responses compared to ADP-Hep (Maubach et al. 2024, Tang et al. 2024).

In *H. pylori*, ADP-Hep—not HBP—has been identified as the primary pathogen-associated molecular pattern (PAMP) (Pfannkuch et al. 2019). Pfannkuch *et al*. employed ultraperformance liquid chromatography-mass spectrometry (UPLC-MS) to demonstrate that *H. pylori* lysates contain only trace amounts of HBP, insufficient to trigger NF-κB activation, whereas ADP-Hep was the predominant immunostimulatory molecule, exhibiting substantially stronger immunological activity than HBP (Pfannkuch et al. 2019). These findings solidify ADP-Hep's role as a key PAMP to induce immune response during *H. pylori* infection. Recent studies indicate that ADP-Hep delivery in *H. pylori* depends on a functional cag-type IV secretion system (cag-T4SS) (Stein et al. 2017, Zimmermann et al. 2017). Further elucidating its immunomodulatory effects, Micheal et al.revealed that the ADP-Hep/ALPK1/TIFAsome/NF-κB signaling axis triggers R-loop formation in gastric epithelial cells, leading to replication stress and DNA damage (Bauer et al. 2020) (Figure 5B). Intriguingly, ADP-Hep also disrupts antigen presentation in macrophages and dendritic cells while activating neutrophils to secrete pro-inflammatory cytokines (TNF-α, IL-1β, IL-8) (Coletta et al. 2021, Faass et al. 2021, Faass et al. 2023, Neuper et al. 2024). Collectively, these mechanisms may enable *H. pylori* to persist within the host by simultaneously evading adaptive immunity and exacerbating inflammation.

Given that ADP-Hep serves dual roles as (1) the essential precursor for LPS heptose biosynthesis and (2) a novel PAMP delivered via T4SS to activate the ALPK1-TIFA immune pathway (Figure 5B), this raises an important mechanistic question: does the absence of DD-heptan in LPS of East Asian *H. pylori* strains, which consequently requires less ADP-Hep for LPS biogenesis compared to DD-heptan-containing Western strains, result in enhanced availability of ADP-Hep for T4SS-mediated delivery to host cells? This warrants further investigation to determine whether the presence or absence of DD-heptan in LPS structure influences the delivery of ADP-Hep to host cells for subsequent activation of the pro-inflammatory ALPK1-TIFA signaling cascade.

## The potential for developing LPS-based conjugate *H. pylori* vaccine

Given that *H. pylori* infection is the leading cause of gastric cancer and with increasing antibiotic resistance, developing an effective vaccine remains a critical unmet medical need. Although multiple vaccine candidates targeting various key bacterial proteins- including urease, OM proteins (e.g. SabA, HpaA, AlpA, OipA), and important virulence factors (e.g. VacA, CagA)—have been explored, no vaccine has yet succeeded (Sutton and Boag 2019, Hasanzadeh Haghighi et al. 2024, Yamaoka 2024). This can be largely attributed to *H. pylori*’s ability to evade immune responses (Sutton and Chionh 2013, Sutton and Boag 2019). As a result, these vaccines did not induce a strong or sustained immune response to effectively protect against *H. pylori* infection or clear existing infections. Notably, earlier *H. pylori* vaccine development efforts primarily aimed to induce a strong Th2 immune response, as this was hypothesized to be particularly effective against *H. pylori*—a pathogen that colonizes mucosal surfaces. However, the accumulating evidence now suggests that a strong Th1 immune response is needed for effective immunization (Sutton and Doidge 2003, Shi et al. 2005, Taylor et al. 2006, Taylor et al. 2008, Oertli et al. 2013). One promising candidate, IMX101, which includes glutamyl-transpeptidase (GGT) as an antigen along with a mucosal adjuvant, has shown potential in countering immune evasion by neutralizing GGT's ability to impair T-cell mediated immunity (Oertli et al. 2013). The GGT-based vaccine elicited robust Th1-type immune responses and has successfully completed Phase I trials (Oertli et al. 2013, Sutton and Boag 2019). Current understanding suggests that an effective *H. pylori* vaccine strategy should target bacterial immune evasion mechanisms while promoting Th1-type immunity. Among the critical immune evasion factors, LPS plays a central role. Notably, previous studies have shown *H. pylori* LPS containing whole cell sonicate induces a robust Th1 response, which enhances bacterial clearance (Ogawa et al. 1997, Sutton and Doidge 2003, Taylor et al. 2006). In contrast, LPS depleted sonicate elicit primarily Th2 responses, underscoring LPS's critical role in driving protective immunity (Taylor et al. 2008). Therefore, an LPS-based glycoconjugate vaccine represents a promising strategy for *H. pylori* vaccine development.

Previous studies have demonstrated that LPS isolated from *H. pylori* strains and conjugated to carrier proteins can elicit functional immune responses, supporting the potential of LPS-based glycoconjugate vaccines (Altman et al. 2012, Monteiro et al. 2011, Altman et al. 2014). Currently, the most common method for LPS isolation from bacterial cultures is the hot-phenol extraction, a process that is not only time-consuming and labor-intensive but also suffers from low yield efficiency. Furthermore, contamination by lipoproteins, nucleic acids (DNA/RNA), and other cellular components in the preparations may confound downstream evaluations of LPS-based glycoconjugate vaccines. These limitations highlight the need for optimized, industrial-scale LPS production methods (Li et al. 2024).

For LPS-based glycoconjugate vaccines derived from other Gram-negative bacteria, standard protocols require the removal or chemical detoxification (e.g. alkaline/acid treatment) of lipid A to eliminate its endotoxic activity. However, *H. pylori* LPS exhibits inherently low endotoxicity due to its unique, constitutively modified lipid A structure. This characteristic may obviate the need for harsh detoxification steps (e.g. detergent treatment) (Li et al. 2024), thereby preserving lipid A's intrinsic adjuvant properties to enhance vaccine immunogenicity.

A critical consideration for *H. pylori* LPS vaccines is the removal of Lewis antigens, which mimic host blood-group antigens and could potentially trigger autoimmune responses. This can be readily achieved through genetic engineering—for example, by deleting glycosyltransferase genes (e.g. *HP0826*, encoding the galactosyltransferase essential for Lewis antigen biosynthesis) (Li et al. 2024).

Considering the heterogeneous LPS isolated from bacterial cultures, recent efforts have focused on the chemical synthesis of well-defined *H. pylori* LPS carbohydrate structures. Ying and colleagues have synthesized several *H. pylori* glycan motifs, including an outer core octasaccharide (Zou et al. 2018), a panel of different length of glucan structure (Tian et al. 2020), the serotype O6 tridecasaccharide (comprising a terminal Ley tetrasaccharide, a heptose pentamer, and a Gal and three Hep residues) (Tian et al. 2020), and a core undecasaccharide (Zou et al. 2022). However, it should be noted that these synthetic structures were based on earlier structural characterizations. Our comprehensive structural reanalysis reveals that the core oligosaccharide and Trio structures are both highly conserved across *H. pylori* strains and essential for bacterial colonization (Li et al. 2017, Li et al. 2019). Based on these findings, we have chemically synthesized a precisely defined nonasaccharide incorporating these key motifs—the conserved core hexasaccharide and Trio trisaccharide (Li et al. 2024). This synthetic antigen is currently being conjugated to carrier proteins for systematic evaluation of its immunological properties and potential as a vaccine candidate against *H. pylori* infection.

## Concluding remarks and future perspectives

*H. pylori* LPS is a critical virulence factor, with its distinctive structure playing essential roles in immune evasion, chronic infection, and pathogenesis. Recent research has significantly advanced our understanding of *H. pylori* LPS, revealing key structural and functional features. The lipid A moiety undergoes constitutive modifications, adopting a dephosphorylated, tetra-acylated, mono-Kdo configuration that confers resistance to host antimicrobial peptides and reduces TLR4 activation. The core oligosaccharide, now recognized as a short, conserved hexasaccharide, lacks the typical inner and outer core organization, while the O-antigen is much longer than previously thought, encompassing Lewis antigens, Trio, DD-heptan, and α-1,6-glucan.

Comparative genomic and animal colonization studies demonstrate that the conserved core and Trio structures are essential for bacterial colonization. Lewis antigen mimicry serves as a double-edged sword, enabling immune evasion while potentially triggering autoimmune responses. Notably, the presence or absence of DD-heptan distinguishes Western and East Asian strains, suggesting a possible link to regional disease outcomes. Furthermore, ADP-heptose, a precursor for LPS heptose residues, has been identified as a novel PAMP that triggers the pro-inflammatory ALPK1-TIFA axis. Future studies should investigate whether DD-heptan influences ADP-heptose delivery and subsequent host immune responses.

LPS-based glycoconjugate vaccines represents a promising strategy for *H. pylori* prevention. Advances in LPS isolation, chemical synthesis, and conjugation techniques including click chemistry or in vivo protein glycan coupling technology, may accelerate vaccine design and development. Further research should explore how strain-specific LPS variations influence host-pathogen interactions and disease progression, paving the way for targeted therapeutics and improved vaccine candidates. A deeper understanding of LPS-mediated immune modulation will be crucial for combating *H. pylori* infections and associated complications.

## Highlights

*H. pylori* LPS displays a unique lipid A structure that undergoes a five-step enzymatic modification, which results in reduced immunogenicity.

Structural studies revealed the redefinition of core oligosaccharide and O-antigen regions and glycosyltransferases involved in *H. pylori* LPS biosynthesis.

The conserved core oligosaccharide and Trio domains of LPS demonstrated essential roles for *H. pylori* colonization.

The Lewis antigen of *H. pylori* LPS is a double-edged sword of immune evasion and autoimmunity.

Comparative genomic studies unraveled the absence of heptan being the most significant LPS variation in East Asian *H. pylori* strains compared to Western *H. pylori* strains, which may promote gastric cancer development through its donor, ADP-heptose in East Asia.

LPS-based vaccines is promising candicates controlling *H. pylori* infection.
